# NutriBase – management system for the integration and interoperability of food- and nutrition-related data and knowledge

**DOI:** 10.3389/fnut.2024.1503389

**Published:** 2025-01-06

**Authors:** Eva Valenčič, Emma Beckett, Tamara Bucher, Clare E. Collins, Barbara Koroušić Seljak

**Affiliations:** ^1^Computer Systems Department, Jožef Stefan Institute, Ljubljana, Slovenia; ^2^Jožef Stefan International Postgraduate School, Ljubljana, Slovenia; ^3^School of Health Sciences, College of Health, Medicine and Wellbeing, University of Newcastle, Newcastle, NSW, Australia; ^4^Food and Nutrition Research Program, Hunter Medical Research Institute, Newcastle, NSW, Australia; ^5^School of Environmental and Life Sciences, College of Engineering, Science and Environment, University of Newcastle, Newcastle, NSW, Australia; ^6^Department of Science, Nutrition Research Australia, Sydney, NSW, Australia

**Keywords:** database management system, food data compilation, food composition data, food composition database, knowledge base

## Abstract

**Introduction:**

Contemporary data and knowledge management and exploration are challenging due to regular releases, updates, and different types and formats. In the food and nutrition domain, solutions for integrating such data and knowledge with respect to the FAIR (Findability, Accessibility, Interoperability, and Reusability) principles are still lacking.

**Methods:**

To address this issue, we have developed a data and knowledge management system called NutriBase, which supports the compilation of a food composition database and its integration with evidence-based knowledge. This research is a novel contribution because it allows for the interconnection and complementation of food composition data with knowledge and takes what has been done in the past a step further by enabling the integration of knowledge. NutriBase focuses on two important challenges; data (semantic) harmonization by using the existing ontologies, and reducing missing data by semi-automatic data imputation made from conflating with existing databases.

**Results and discussion:**

The developed web-based tool is highly modifiable and can be further customized to meet national or international requirements. It can help create and maintain the quality management system needed to assure data quality. Newly generated data and knowledge can continuously be added, as interoperability with other systems is enabled. The tool is intended for use by domain experts, food compilers, and researchers who can add and edit food-relevant data and knowledge. However, the tool is also accessible to food manufacturers, who can regularly update information about their products and thus give consumers access to current data. Moreover, the traceability of the data and knowledge provenance allows the compilation of a trustworthy management system. The system is designed to allow easy integration of data from different sources, which enables data borrowing and reduction of missing data. In this paper, the feasibility of NutriBase is demonstrated on Slovenian food-related data and knowledge, which is further linked with international resources. Outputs such as matched food components and food classifications have been integrated into semantic resources that are currently under development in various international projects.

## 1 Introduction

Food and nutrition-related data and knowledge (D&K) are essential for many research domains, including public health surveillance and promotion, dietary and health assessments, disease prevention, nutrition education, consumer protection, agriculture, food policy, and food labeling (1, 2). D&K, such as food composition data or dietary guidelines, are also necessary for stakeholders in the food industry, retail sector, non-government organisations, policymakers, and ultimately consumers. Consumers rely on D&K when making food and nutrition decisions, while policymakers use food and nutrition-related D&K to obtain accurate scientific evidence needed to design and promote strategies required to improve public health and overall well-being (3, 4).

However, D&K are complex, covering diverse areas such as food composition, food safety, food authenticity, and consumption. This paper focuses on food composition data (FCD) and knowledge for dietary assessment and advising. This is highly important for domain experts and policymakers, as well as consumers, including patients. While FCD contains detailed compositional, biochemical, and physiological data of foods (e.g., how much vitamin C apples contain), *knowledge* provides additional food-related information (e.g., what is the recommended intake of vitamin C). FCD and knowledge are compiled in various databases; however, their integration and interoperability are lacking (5). Improved integration would enable easier access the latest evidence-based D&K from different research areas within a single system.

Nowadays, FCD is compiled online in the form of a food composition database (FCDB). FCDBs are usually compiled at the national level but are often used internationally to conduct public health studies (2). Examples include multiple European FCDBs [available through the FoodEXplorer tool (6)], USDA’s FoodData Central (7), FAO/INFOODS databases (8), Canadian FooDB (9), and others. In general, FCDBs contain data on traditional, ethnic, and local foods and dishes, with some combining generic and branded foods [e.g., Serbian (10)] and others maintaining separate databases for different food types [e.g., Dutch branded food database (11)]. In addition to institutional databases, numerous company-owned FCDBs also exist, such as the Edamam’s food, grocery, and (restaurant) database composed using Natural Language Processing (NLP) techniques (12) and GS1 branded foods, and barcode databases maintained through the Global Data Synchronization Network (GDSN) (13).

There are two main challenges with existing FCDBs. Namely, data harmonization and missing data. First, FCBDs may contain data of different quality due to differences in data production methods (food sampling, analyses or estimation, (re)calculation, borrowing), data compilation (collection, aggregation, compilation, and dissemination), and data management. The challenge of data harmonization has been addressed by several networks of excellence. For example, the Food CEN standard (14), which defines requirements on the structure and semantics of food datasets and of interchange of food data. Another initiative, the ESFRI research infrastructure Metrofood (15), contributes to the development of aligned metrology services in the food domain. Moreover, when compiling a FCDB, guidelines and frameworks to assess the quality of data, datasets, and databases (16, 17) need to be acknowledged. Several frameworks also enable unified data classification and description, which need to be considered when harmonizing various FCDB (2, 18, 19). While these standards and frameworks facilitate the harmonization of food- and nutrition-related data, the problem of linking it with other data types (e.g., medical, environmental, and consumption-related) remains unresolved. The second challenge is related to missing data in FCDBs, which distorts data integrity. Analyzing all components of specific foods poses a significant financial burden for institutions; thus, no FCDB is complete, and updates are not done continuously. The challenge of missing FCD is being addressed in various ways, including borrowing data from other databases, performing tedious manual work, or using computer-supported methods for (semi-) automated data imputation (20, 21).

On the other hand, together with databases, knowledge bases (KBs) are also very important resources. By definition, a KB is an easily accessible online library of collected and organized information and documentation about certain topics (22). The important knowledge that should be included in food and nutrition KB should include, but not be limited to: standardized classification and description of coding systems [e.g., LanguaL (23), FoodEx2 (24), INFOODS (8)]; standardized value documentation (e.g., acquisition type, method type) (18); a chemical databases of molecular entities – ChEBI (25); retention and yield factors used to calculate the nutrient content of composite dishes or recipes (26); standardized household measurement units; national dietary reference values and dietary guidelines; physical activity standards; food components’ bioavailability; food-drug interactions, and others.

As knowledge accumulates quickly, the creation and maintenance of a KB is tedious work, usually done manually by domain experts. However, semantic resources have complemented KBs and allowed interoperability of D&K from various research domains. Semantic resources like the ontologies [e.g., FoodOn (27), ISO-FOOD (28), FNS-Harmony (29), COMFOCUS (30)] or knowledge graphs [e.g., describing complex relationships between food and biomedical factors (31)] are being developed to formally describe knowledge as a set of concepts and the relationships between those concepts within a domain. To link FCD with semantic resources, FCD needs to be annotated with standardized metadata in machine-readable formats to enable connectivity of terms across different data sources.

Regardless of all research efforts, applicable KBs providing integrated knowledge on food and nutrition are still lacking. There are few KBs that focus on specific subdomains, such as FoodKG (32) for food recommendation based on diet-related knowledge or TasteAtlas (33), a world atlas of traditional dishes, local ingredients, and authentic restaurants.

The food and nutrition community has created many FCDBs as well as few KBs, but their integration and interoperability are currently missing. Even when limited just to the integration within FCDB, information is not harmonized because different coding systems, documentation or standards are used. Some examples of best practice using harmonized FCD are FoodEXplorer (6), FoodCASE (34), FoodData Central, Glycemic Index Research and GI News (35). Some of these tools even enable comparison of FCD from multiple countries. This is important as, with increasing globalization, the availability of international foods and dishes is increasing, and obtaining datasets of non-local foods is necessary. Having databases composed on a national level is important; however, for applied science, it would be useful if compilers could link and integrate not only FCD with each other but also FCDBs with KBs. This is something that we believe does not yet exist or is not publicly available in the food and nutrition domain. The importance of integration and interoperability was also highlighted in the recent paper by Durazzo et al. (36), which further emphasized the necessity of cooperation and D&K sharing between compilers. However, the connectivity among computer systems and/or online platforms is equally necessary.

In the current paper, we introduce a new database management system, called NutriBase, for integrating FCD from different databases with food- and nutrition-related knowledge. The integration is performed in a transparent way and enables, together with harmonization, a reduction in missing data. In Section 2, we explain how publicly available D&K resources, which (currently) represent the baseline of the NutriBase, were identified and collected. Next, we introduce NutriBase and describe its functionality. In Section 3, we describe the compilation process of the Slovenian FCDB and KB, identify issues, discuss possible solutions the system offers, and provide plans for future work. We conclude the paper in Section 4.

## 2 Materials and methods

### 2.1 Data and knowledge collection

To demonstrate the feasibility of NutriBase, Slovenian FCD and both, national and international semantic resources were collected. Firstly, the analytical compositional data on generic foods from the Slovenian FCDB composed in 2006 and updated in 2012 (37) were imported. The recipes included in the Slovenian FCDB were imported separately, as they require different data handling, such as consideration of yield and retention factors, as well as standards for calculating recipes (38, 39). In addition, branded foods that can currently be purchased in Slovenia, are being uploaded through an application programming interface (API) from the Composition and Labeling Information System (CLAS) (40).

To complement the Slovenian FCDB for generic foods, six publicly available FCDBs (Table 1), together with associated metadata and documentation, were either downloaded or linked through an API in late 2020 or 2021. The imported FCDBs consisted of datasets in different formats, and not all of them adhered to the Food CEN standard (14). The imported metadata and documentation include various background information, such as explanations of data sources, procedures for data quality assurance, descriptions of foods and food group classification levels, and explanations of specific component descriptions, calculations and units used. Multiple foreign FCDBs needed to be imported because they contain different data. For example, FoodData Central (US in Table 2) in addition to FCD, provides also the data for household measurement units (e.g., tablespoon, cup, dash) which can be linked to generic foods. Moreover, different components are collected or analyzed across different FCDBs. For instance, some datasets contain data for total carbohydrates (digestible and indigestible, including dietary fiber), whereas others contain only data for available carbohydrates. From the currently imported FCDBs only three provide data for total carbohydrate, however all of them contain data for available carbohydrates and total dietary fiber, thus the total carbohydrates could be calculated. Lastly, relevant evidence-based food and nutrition knowledge was systematically reviewed and collected from publicly available national and international resources, and was further compiled into the NutriBase KB (Table 2).

**Table 1:** FCDBs currently included in the NutriBase.

Currently Imported FCDBs				
Country code	No. of components	No. of food group levels^‡^	No. of foods / dishes	Source file format
SI	773*	15	993	.CSV/.XSL
		48		
		149		
FR	60	10	2,807	.CSV/.XSL
		58		
		83		
NL	133	27	2,152	.CSV/.XSL
DK	197	18	1,186	.CSV/.XSL
		127		
UK	178	14	2,910	.CSV/.XSL
		71		
		54		
AU	249	22	1,534	.CSV/.XSL
		97		
US	235	28	7,793^†^, 210¨	API

*651 from EuroFIR Thesauri document and 122 subsequently added (own); ‡ = the top number is the highest level, the bottom number is the lowest (the most detailed) level (sub-level); † = SR Legacy Foods; ¨ = Foundation Foods.

**Table 2:** Resources included in the NutriBase KB.

Scemantic resources			
Resource name/type and reference	Knowledge type	Description	Number of entities
Standardized classifications and description coding system	FoodEx2 classification	A food classification and description system developed by EFSA - includes different hierarchies and facets for different food safety domains. (e.g., A00KR#F27.A00KV$F27.A00LN $F27.A00LB$F27.A00LG; mixed leafy vegetables)	4,445
Standardized value documentation (11)	Component type	Component identifiers and descriptors (e.g., CHO; carbohydrate; use for total of those carbohydrates digested and absorbed in the intestine; total accessible carbohydrates include free sugars, polyols and dextrins, starch, and glycogen).	660 (9 of these are for backward compatibility only) 56 classification identifiers (not used for new indexing)
	Unit	E.g., grams, millimoles, alpha- tocopherol equivalent, per cent.	19 Additional 20 added (IU, g/kg body mass, etc.)
	Matrix unit	E.g., per 100 g of total food, per 100 mL food volume, per unit, per 100 g edible portion.	20 matrixes
	Value type	E.g., arithmetic mean, best estimate, average, below limit of detection, trace.	20 types
	Method type	Reporting if the value was analyzed, calculated or imputed (e.g., calculated as recipes, calculated from related food, analytical result).	20 types
	Method indicator	Providing details for the analytical method or formulas used for calculation (e.g., chromatography, difference, ash calculated as sum of minerals).	214 indicators
	Acquisition type	Describes the origin of the value (e.g., laboratory, food composition table, authoritative document).	12 types
	Reference type	E.g., article in journal, file or database, product label, software.	14 types
LanguaL thesaurus (10)	Cooking methods	E.g., griddled, cooked by microwave, deep fried.	47 methods
FoodData Central at US Department of Agriculture (USDA)	Measurement and household units	E.g., tea spoon, slice, filet, cup, could be used for volume to weight conversions.	115 (currently in use) out of 1923
ChEBI - a chemical database and ontology of molecular entities, which is part of the Open biomedical ontologies at the EBI, and European ELIXIR infrastructure	Dictionary of molecular entities	Providing detailed data of chemical entities of biological interest (e.g., definitions, formulas, ontologies, chemical reactions, IUPAC names and identifiers)	210 linked to added components
SciName Finder (26)	Search tool for scientific and common names of plants and animals	Providing precise identify plants and animals Allows precise identification of plants and animals, and searching the information on scientific and common names provided by authoritative resources (and not from secondary sources)	More than 1,000,000 scientific and common names
Culinary groups [adapted from (18, 23)]	Culinary groups / subgroups related to retention and yield factors.	Providing the basics for obtaining nutrient content of foods by calculation methods (as recipe calculation), based on the amount of ingredients given in a recipe, nutrient composition of ingredients and factors that consider changes in nutrient content (retention factors), and weight (yield factors) during preparation.	31 groups and subgroups related to yield factors, and 38 related to retention factors
Slovenian dietary reference values (DRVs) (27) based on the D-A-CH reference values adopted by the Ministry of Health of the Republic of Slovenia	DRVs	Reference values for energy and nutrient intake for children (at least 1-year old), adolescents, adults, elderly, pregnant women and nursing mothers.	34 references for energy, macro- and micronutrients, for men and women (10 different age groups)
Latest dietary guidelines and recommendations	National and international dietary guidelines and recommendations	Relevant evidence-based guidelines and recommendations for different consumers (athletes, pregnant women and nursing mothers, healthy individuals from different age groups).	Currently defined for biomarkers (blood cholesterol and glucose) and endurance sports.
Physical activity related standards	Metabolic equivalent of task (METS)	E.g., basketball, swimming, mopping, walking, sitting.	541 tasks
	Physical activity level (PAL)	E.g., sedentary or light activity lifestyle.	5 levels per sex

The approaches and tools applied and described in the current paper can be used for D&K from any country. The Slovenian D&K are used as an example only. Unlimited publicly available FCDBs and/or KBs can be uploaded or linked via an API to create a new database, as long as they comply with the NutriBase requirements.

### 2.2 NutriBase - data- and knowledge base management system

**Figure 1:**
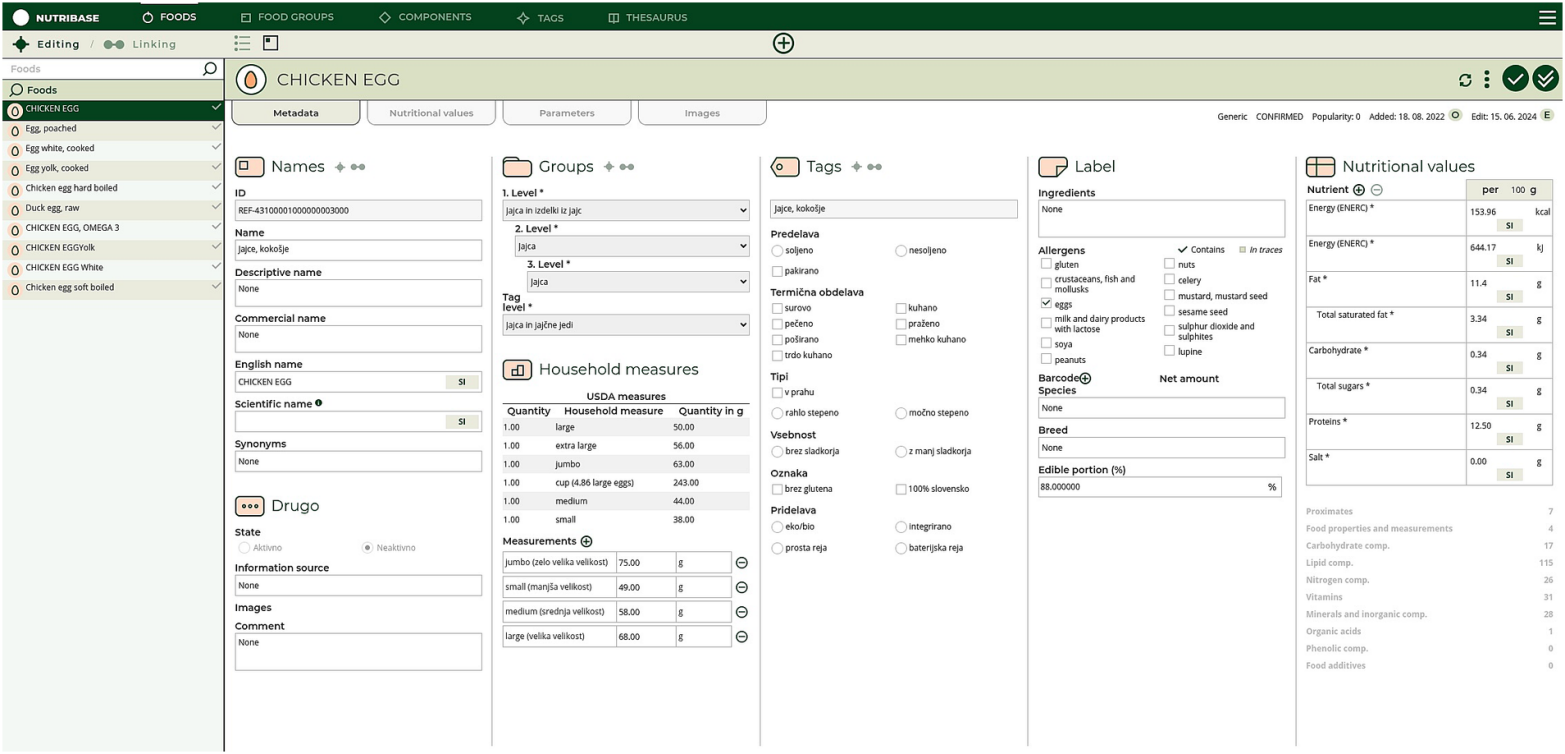
User interface of NutriBase.

NutriBase is designed to enable easy integration with other KBs and semantic resources conceptualizing the health, environmental, consumer behaviors, and food and nutrition domains in particular. This data- and knowledge base management system (DKBMS) has been implemented as a web-based tool (Figure 1) for food compilers to easily explore, compile and most importantly, link data from different FCDBs and KBs. The main goal of this process is achieving an optimal linking of D&K, which enables borrowing data respecting the FAIR (Findability, Accessibility, Interoperability, and Reusability) principles for data management (41), and reducing missing D&K.

#### 2.2.1 FCDB compilation

**Figure 2:**
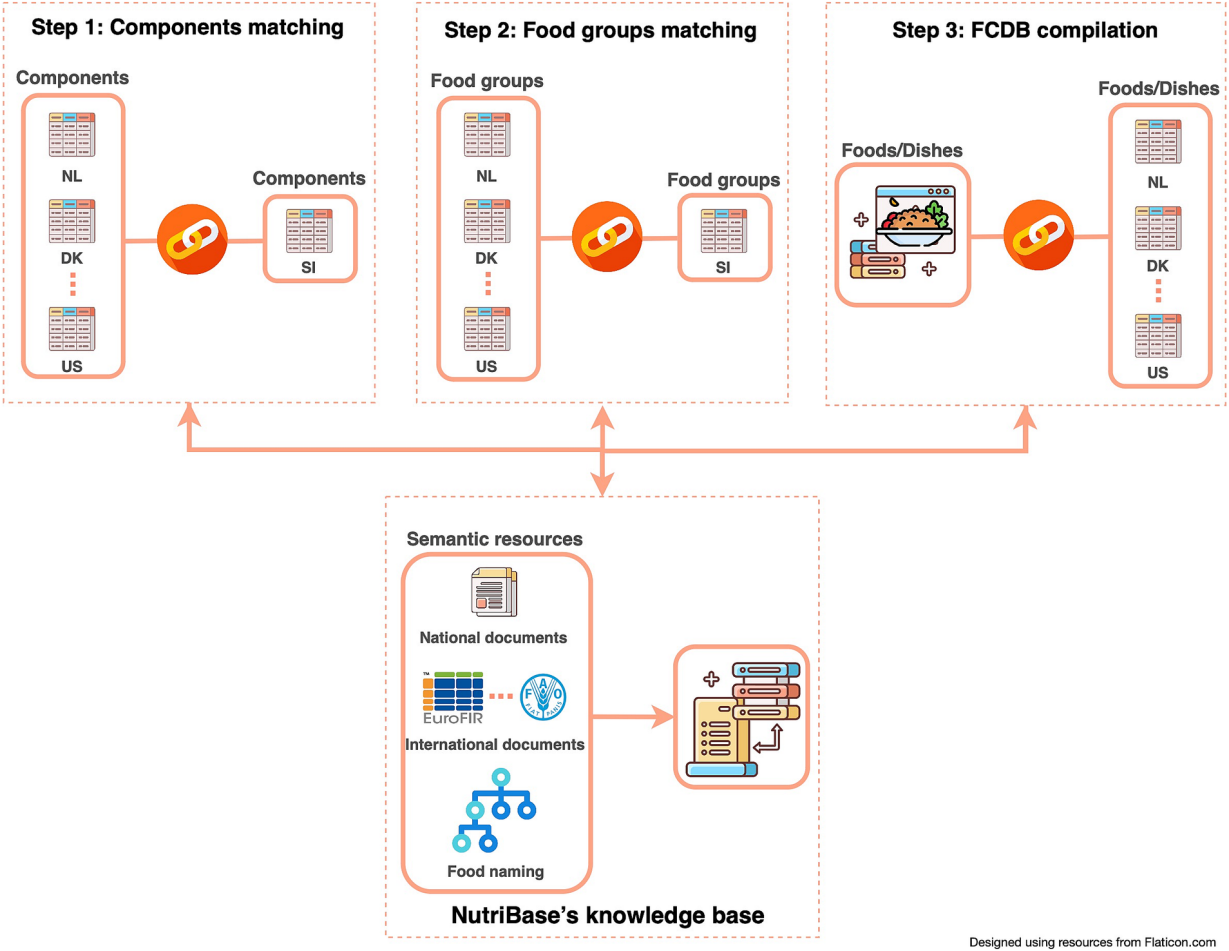
Flowchart of compilation process to link foods from different FCDBs.

To ensure a semi-automatic connectivity among different sources (FCDBs), standardized components and food groups matching had to be manually performed (Figure 2; Step 1 and 2). Since the composition of food depends on its geographical origin, it is important to also consider the data source and the data most closely related to local foods. Therefore, a pre-set priority list of data sources is integrated within the system and can be adapted if needed. For Slovenian example this means that European datasets are prioritized before non-EU datasets. This allows experts to semi-automatically compile datasets that are as complete as possible, while also transparently providing the source of specific data (e.g., component value). The pre-set priority list can easily be amended or set for different countries. Moreover, a comparison of a national dataset (in our case, Slovenian), with other, foreign datasets is also enabled. This feature allows borrowing specific data from other FCDBs. Together with food composition data, compilers can also check additional value information, such as value type and method type (if provided). Being able to check additional value information and standards, allows compilers to assess the quality of the data and select the most appropriate or accurate one. Additionally, during the FCDB compilation process, basic food information and metadata, such as generic and/or commercial names, allergens, ingredients, food origin, and food images, are also addressed and can be borrowed.

NutriBase presents an infrastructure that can be adapted for FCD from any country. However, to achieve an optimal linking of D&K and to ease and expedite FCDB compilation, various knowledge resources had to be considered.

#### 2.2.2 Knowledge base compilation

In the NutriBase underlying thesaurus, knowledge about relevant food- and nutrition topics is collected and maintained. The KB, implemented within the DKBMS, is connected with all three steps of the workflow seen on Figure 2. Thus, all updates of the KB content will have an immediate impact on linked data in FCDB. That means whenever a new data or knowledge is published, it can easily be imported and linked to existing D&K or substituted for the latest findings. An important part of the implemented KB is food naming by using tags. It provides functionality for unique food naming and metadata annotation. While much work has already been conducted on unifying food description and classification, food naming is still an open issue. Therefore, we have implemented a new food-tagging approach to unify and standardize food naming within the FCDB. This is especially useful when different users are working on a FCDB, as it enables unambiguous communication between all users involved in the working process. In addition, together with using tags, setting rules for food naming has been proposed as another solution.

#### 2.2.3 Usability of NutriBase

Lastly, the usability of the newly developed system was evaluated. We distributed the System Usability Scale (SUS) questionnaire among regular NutriBase users with different profile roles. The SUS tool is a reliable and validated tool for measuring the usability, which is frequently used by evaluators of mHealth services (42). It consists of a 10-item questionnaire with five response options for respondents (strongly agree to strongly disagree). The survey was completely anonymous and after collecting the responses, the participant’s scores were carefully interpreted to produce a percentile ranking.

## 3 Results and discussion

### 3.1 The compilation of the Slovenian FCDB and KB

**Figure 3:**
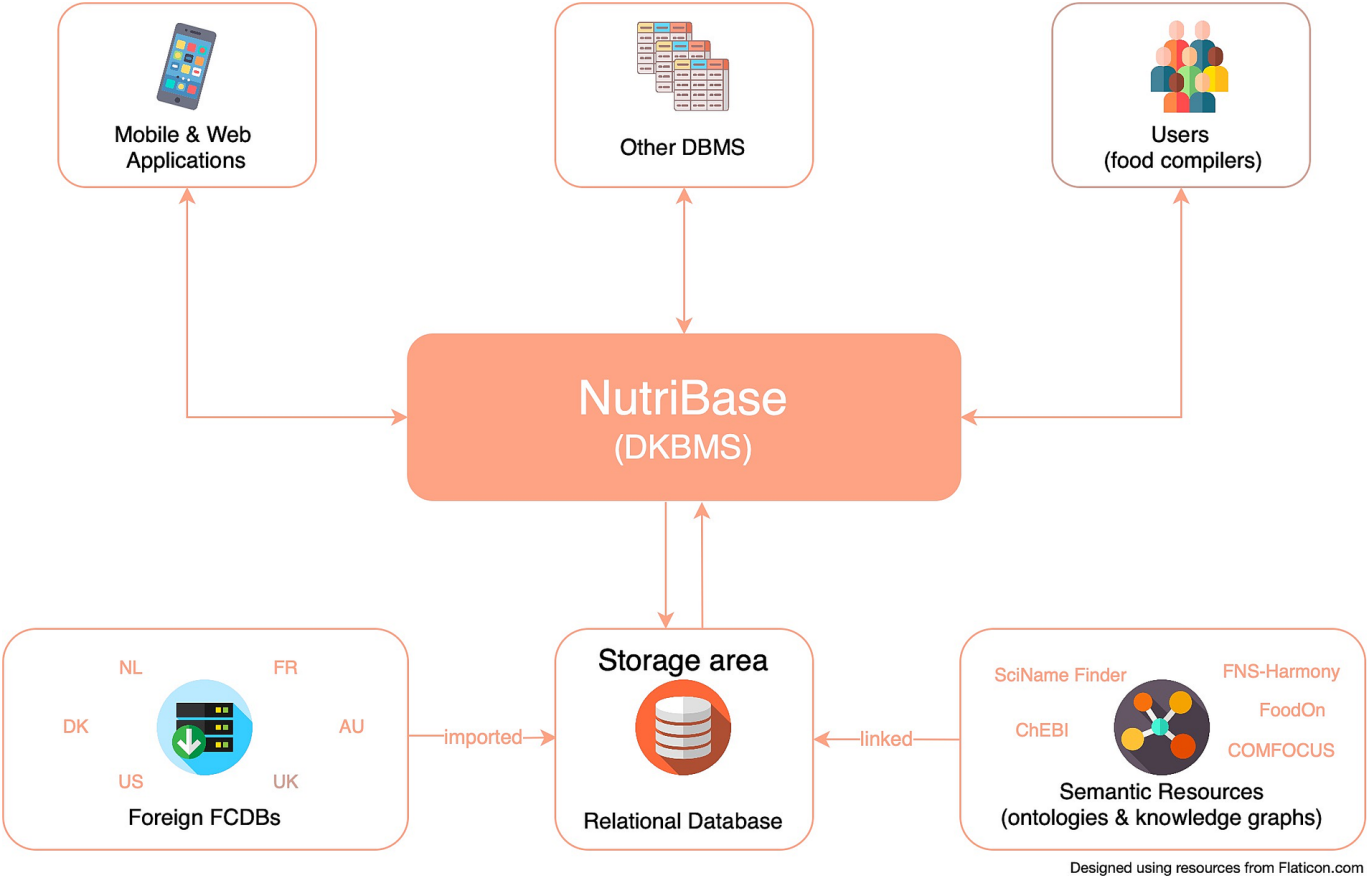
Overview of NutriBase structure.

Throughout the entire compilation process (Figure 2), D&K were maintained in accordance with the FAIR principles. Managing D&K to ensure that the format of foreign FCDBs and KBs remains unchanged from the original sources has been a key requirement in NutriBase’s development (Figure 3).

#### 3.1.1 Components matching

**Figure 4:**
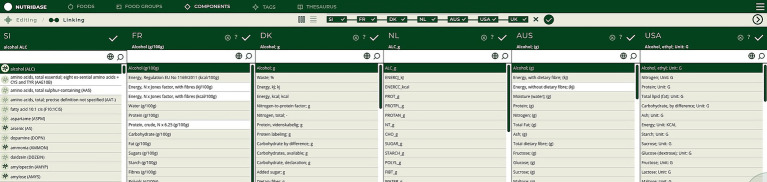
User interface of component matching process.

To create and link the Slovenian database, the compilation process was initiated by components matching (Figure 2, Step 1). The Slovenian FCDB complies with the CEN Food standard (14), therefore the components specified with respect to the EuroFIR thesaurus for components (18) were manually matched with components from the foreign FCDBs (Figure 4 presents the user interface of this process). Although most of the foreign selected FCDBs also comply with the CEN Food standard, mismatched components (i.e., different names for the same components among different countries) were still present (examples are shown in Table 3).

**Table 3:** An example of component matching of Slovenian components with components from foreign datasets.

Component names among different FCDBs						
SI	FR	NL	DK	UK	AU	US
Carbohydrate, total (CHOT)	/	/	Carbohydrate by difference; g	/	/	Carbohydrate, by difference; Unit: G
Carbohydrate (CHO)	Carbohydrate (g/100 g)	CHO g	Carbohydrates, available; g, Carbohydrate, declaration; g	Carbohydrate (g); CHO	Available carbohydrate, with sugar alcohols; (g)	Carbohydrate, by summation; Unit: G
Fiber, total dietary (FIBT)	Fibers (g/100 g)	FIBT_g	Dietary fiber; g	AOAC fiber (g); AOACFIB	Total dietary fiber; (g)	Total dietary fiber (AOAC 2011.25); Unit: G, Fiber, total dietary; Unit: G
Fat, total (FAT)	Fat (g/100 g)	FAT_g	Fat, total; g	Fat (g); FAT	Total Fat; (g)	Total lipid (fat); Unit: G
Fatty acids, total saturated (FASAT)	FA saturated (g/100 g)	FASAT_g	Sum saturated; g	Satd FA /100 g FA (g); SATFAC, Satd FA /100 g (g); SATFOD	Total saturated fatty acids;(%), Total saturated fatty acids; (g)	Fatty acids, total saturated; Unit: G

Components were matched manually by domain experts to ensure a correct and unambiguous matching. Moreover, the result can be provided as an input to the FNS-Harmony ontology (43), which has been developed within the FNS-Cloud project to support interoperability of food- and nutrition-related data in the European Open Science Cloud (EOSC) and is available through the NCBO Bioportal. NutriBase could be integrated with FNS-Harmony, which reuses or incorporates several ontologies, including FoodOn (27). In this case, food compilers would not only be able to provide but also use new knowledge about semantic integration with other systems, such as GS1 GDSN (44).

#### 3.1.2 Food groups matching

Firstly, food groups were designed based on the classification of foods used by relevant information systems in Slovenia, as well as the EuroFIR standard (18), which is intended for generic foods. Since Slovenian FCDB also includes branded foods, classification systems for these had to be considered as well. However, we found that different Slovenian institutions use different classification systems. This suggests that even within a single country, it might be necessary to follow and comply with several standards. For example, the Slovenian classification system, which is based on public procurement and is determined by law, or the Dunford classification system (45), specifically developed for branded foods. Currently, the Slovenian FCDB includes three hierarchical classification levels: 15 groups on the first, 48 groups on the second, and 160 on the third (and most detailed) level.

In addition to manually matching national food classification systems with one another, the food groups used in Slovenian FCDB were also matched with those used in the foreign FCDBs (Figure 2, Step 2). An example of a matched food group - *Fresh vegetables,* among FCDBs is presented in Table 4. The task of food groups matching was especially challenging, as different countries use different numbers of classification levels. For example, foods in France and the UK are classified into up to three levels, in Australia and Denmark into two levels, and in the Netherlands and USA into just one level. Moreover, the level of detail within food groups varies. As shown in Table 4, some countries group all vegetables together, while the others sub-classify them further (e.g., root vegetables, fruiting vegetables).

**Table 4:** An example of matching one Slovenian food group with different foreign FCDBs.

	Classification levels			
		L1	L2	L3
FCBD	SI	Vegetables	Vegetables, mushrooms and algae	Fresh vegetables
	FR	Fruits, vegetables, legumes and nuts	Vegetables	Vegetables, raw
				Vegetables, cooked
	NL	Vegetables	/	/
	DK	Vegetables and vegetable products	Leaf and stem vegetables	/
			Root and tuber vegetables	
			“Fruit” vegetables	
			“Fruit” vegetables	
	UK	Vegetables	Vegetables, general	/
	AU	Vegetable products and dishes	Wild harvested vegetables, and vegetable dishes	/
			Cabbage, cauliflower and similar brassica vegetables	
			Carrot and similar root vegetables	
			Leaf and stalk	
			Tomato and tomato products	
			Other fruiting vegetables	
			Other vegetables and vegetable combinations	
	US	Vegetables and Vegetable Products	/	/

L1, level 1 (the highest level in the hierarchy); L2, level 2; L3, level 3 (the lowest level in the hierarchy).

To ensure accurate food classification and assist users in using NutriBase, a feature was implemented allowing compilers to add examples of foods allocated to specific food group. This feature was found to be very useful, as it enables users to unambiguously select the correct food group. Additionally, manually matched food groups can also be provided as inputs into FNS-Harmony.

#### 3.1.3 FCDB compilation

FCDB compilation process (Step 3 in Figure 2) began with manually checking and correcting a dataset of 14,064 entries for 443 generic foods analyzed by the Biotechnical Faculty of the University of Ljubljana in 2006 and 2012 (37). Together with the composition data, annotated metadata (e.g., value information) were also reviewed. Certain components were specifically checked to ensure compliance with the standards. For example, the differences between total available carbohydrates and total carbohydrates. This entire process aligns with the first 12 steps of the generic compilation process described by Westenbrink et al. (2), currently excluding Step 5 (attribution of quality index) and Step 11 (physical storage). The evaluation of Slovenian data quality (17) and the database quality evaluation, as suggested by the recently published FAO/INFOODS framework (16), are currently underway.

Next, the Slovenian name for each generic food was reviewed, and a scientific name (when appropriate), an English name, and synonyms were assigned based on the new food-tagging approach. To achieve this, tags were defined, and rules for their application were established within each food group. During this process, we found that similar foods might have different names. This can make searching for a specific food within the FCDB harder for compilers as well as for consumers accessing publicly available FCDBs. For example, the only difference between ‘Baked eggplant with added cheese and tomato sauce’ and ‘Aubergine prepared in tomato sauce and cheese, frozen’ is that one is baked and the other is frozen, but the names are very different. Therefore, using tags for food naming, helps unify the FCDB and simplifies searching for specific foods. Moreover, we ensured the naming is clear to all users, specifically for consumers accessing FCD (e.g., via a mobile app), who may find it challenging to understand the processing conditions of foods. For example, meat can be analyzed as raw (e.g., beef filet) or heat-treated (e.g., beef filet, grilled). However, experience shows, it is seen that consumers do not consider ‘beef filet’ as raw, but rather as ready-to-eat steak. Therefore, adding the ‘raw’ tag to raw meat seemed reasonable. On the other hand, it is clear to consumers that ‘banana’ is raw, and they do not expect this tag added to fresh fruits. Thus, the ‘raw’ tag is used in some food groups but not in others. In addition, the tag ‘peeled’ is used only when appropriate (e.g., ‘apple, peeled’, but not ‘banana, peeled’). Currently, each food group at the third hierarchical level within the tool has an average of 15.4 tags.

Additionally, the initial Slovenian dataset of generic foods was manually linked with the same or similar food items from the selected foreign FCDBs. The linking was carried out by domain experts. First, the English names were compared, followed by a comparison of the main food components. In case the food composition was similar, the food items were linked together and the missing data were imputed from the foreign FCDBs. Table 5 presents an example of the number of imputed data for *Fresh vegetables* food group from a specific FCDB. As can be seen, only one value for total carbohydrates could be borrowed from US database, while the rest were taken from Slovenian FCDB. However, cystine values are missing in Slovenian FCDB, so they were borrowed from the Danish and US databases (the other FCDBs do not contain data for cystine). The NutriBase allows linking one food with multiple foods within one database or across multiple databases. For example, the Slovenian ‘average white bread’ can be linked with ‘white baguette’ and ‘white loaf’ from one FCDB, and with ‘white bread’ from the other FCDBs. The borrowed data will, however, be displayed based on the pre-set priority list of FCDBs. In our case, when a food item is linked with food item(s) from across different FCDBs, data from European datasets were prioritized before non-EU datasets. However, compilers can manually change the data source and select (borrow) non-EU data to be displayed if it is more appropriate. We found this approach to be very convenient, as it provides compilers with data most closely related to the local foods, but it still gives them freedom to select another data. Moreover, the manually matched foods present a valuable asset that can be used to construct a gold standard corpus, i.e., a corpus of text annotated with food entities required for NLP techniques, such as CafeteriaFCD (46).

**Table 5:** Number of data imputed from a specific FCDB for *Fresh vegetables* food group.

Component	SI	FR	NL	DK	UK	AU	US
Carbohydrate, total (CHOT)	42	-	-	0	-	-	1
Carbohydrate (CHO)	24	15	3	1	1	0	0
Fiber, total dietary (FIBT)	36	4	0	1	1	0	0
Fat, total (FAT)	42	0	0	0	1	0	0
Fatty acids, total saturated (FASAT)	28	12	1	0	1	0	2
Fatty acids, total monounsaturated (FAMS)	26	12	-	1	1	0	1
Fatty acids, total polyunsaturated (FAPU)	26	12	1	0	1	0	1
Protein (PROT)	42	0	0	0	1	0	0
Energy, gross (ENERA)	5	4	0	0	1	0	0
Energy, total metabolizable (ENERC)	33	1	5	0	0	0	5
Water (WATER)	41	1	0	0	1	0	0
Ash (ASH)	41	1	0	0	-	0	1
Polyols (POLYL)	0	10	22	0	-	-	0
Alcohol (ALC)	0	31	4	2	2	0	1
Sodium (NA)	39	2	0	0	1	0	0
Salt (NACL)	12	31	-	-	-	-	-
Organic acids (OA)	22	3	0	1	-	-	-
Alanine (ALA)	10	-	-	11	-	3	11
Arginine (ARG)	24	-	-	6	-	0	7
Asparagine (ASN)	0	-	-	-	-	-	-
Cysteine (CYSTE)	15	-	-	-	-	-	0
Cystine (CYS)	0	-	-	18	-	-	13
Glutamic acid (GLU)	10	-	-	11	-	3	11
Glutamine (GLN)	10	-	-	-	-	-	0
Histidine (HIS)	23	-	-	7	-	0	7
Isoleucine (ILE)	25	-	-	5	-	0	7
Leucine (LEU)	24	-	-	6	-	0	7
Lysine (LYS)	25	-	-	5	-	0	7
Methionine (MET)	25	-	-	5	-	0	7
Phenylalanine (PHE)	25	-	-	5	-	0	7
Proline (PRO)	10	-	-	11	-	3	11
Serine (SER)	9	-	-	12	-	3	11
Taurine (TAU)	0	-	-	-	-	-	0
Threonine (THR)	24	-	-	6	-	0	7
Tryptophan (TRP)	20	-	-	8	-	2	8
Tyrosine (TYR)	17	-	-	7	-	2	9
Valine (VAL)	24	-	-	6	-	0	7

- = the component is not present in the FCDB.

Same as generic foods, the branded foods can also be linked with similar generic foods from either national FCDB or foreign FCDBs. In this case, the original FCD of a branded food is taken from the nutrition declaration table, while the FCD not provided on the nutrition declaration table (e.g., micronutrients) can be imputed from FCDBs and transparently marked as such. This is especially beneficial when collecting food consumption data for the national food consumption survey. As seen in the EU Menu project, consumers usually provide only the brand or production line of the food item when reporting food intake. For example, instead of reporting consumption of ‘full fat milk’, they reported a producer’s name of such milk. Since the nutrition declaration table usually only provides the information of energy value and six other nutrients, the values of micronutrients are unknown. Thus, branded foods could be linked with generic foods to compose the complete dataset, which would provide the opportunity to more accurately assess food intake of individuals and overall population.

Finally, yet importantly, internationally accepted algorithms to avoid errors were selected and applied to produce aggregated data [e.g., recipe calculations) (Steps 14 and 15 according to Westenbrink et al. (2)]. In addition, the compiled and aggregated data within the NutriBase were verified [and corrected if needed) (Steps 16 and 17, according to Westenbrink et al. (2)] to prevent hazards related to data validation. The majority of the FCD validation has been done manually, however the tool automatically performs consistency checks for some metadata and components (e.g., content of specific component is not larger than 100 g (converted regardless of the unit), the sum of proximities is ≤105 g, value of saturated fatty acids is not larger than value of total fats, etc.). The validated data is then stored and disseminated [Steps 18 to 22, according to Westenbrink et al. (2)].

#### 3.1.4 Knowledge base creation

Using semantic resources, a KB was created to support the optimal food compilation process, as well as for data quality assessment, traceability, calculations and validation. The KB implemented within the NutriBase is meant to be used by domain experts, as it collects the latest scientific evidence and documentation required for data management and data source management. The KB also consists of the reference list and it allows publication metadata to be imported in standardized formats (e.g., bib). These references can be further linked to specific data/information, which allows traceability of data and metadata. Moreover, the information can be edited or added to the existing KB and updated accordingly. For instance, units listed in the EuroFIR value documentation (18) can be supplemented or extended with other units (e.g., IU, ABV) to meet the compilers’ needs, or they can be updated if changes are made to the existing EuroFIR standards.

#### 3.1.5 Linking FCDB with knowledge

Linking FCD from different sources is important, and linking knowledge from various sources is equally crucial. Both types of linking can be performed in NutriBase; however, the system also enables the linking of FCD with knowledge. For instance, a specific component (e.g., vitamin C) can be linked with a relevant dietary recommendations, such as Slovenian DRVs (47). Therefore, within the tool, data (component; vitamin C) was interconnected and complemented with knowledge (dietary requirements for vitamin C), enabling access to combined information in one place. This approach takes what has been done in the past a step further by incorporating knowledge into the system, which can be especially useful for informing and educating consumers (e.g., via mobile apps). Instead of providing consumers or app users with just FCD, the incorporated knowledge can also be provided, which can deliver a more personalized approach. Our work is consistent with previous works (5, 27, 48), with the difference that NutriBase is a practical and applicable tool, whereas the previous works is theory based.

#### 3.1.6 Tool validation

The NutriBase and its functionalities were validated throughout the entire compilation process of the FCDB and KB. Seven experts who regularly use NutriBase evaluated it using the SUS tool, which is used for judging the perceived usability of systems. The SUS score was 78.9, which falls to 85^th^ percentile and corresponds to grade A-. Moreover, six food compilers of different skills have performed various tasks (e.g., component matching, food linking) depending on their user profile role. For example, less skilled compilers have only edited D&K, whereas more experienced compilers performed more demanding tasks. Regardless of their skill level, all users agreed that the system is a helpful, easy-to-use tool when compiling a FCDB, especially because it collects all relevant and needed D&K in one place.

### 3.2 Strengths and limitation of DKBMS

While reviewing analytical data of generic foods from the past Slovenian FCDB and importing it into the DKBMS, some errors and gaps were identified and further discussed with compilers. The data was reviewed using spreadsheets, and it was found that errors were difficult to identify. However, when using NutriBase to review and edit the FCD, users agreed that it is a useful and reliable tool. Although spreadsheets are very popular when handling data, a similar finding was reported by Presser et al. (34).

To assess the quality of D&K, it is crucial to develop and maintain a quality management system (2). Currently available FCDBs contain data of varying quality, mainly due to the use of different resources and different methods of data acquisition. The metadata used to describe them, as well as the quantity of data differ among FCDBs. Therefore, compilers need to follow standardized guidelines, provide quality indexes for their original data, and further evaluate their FCDB. This will help domain experts select the best high-quality dataset and/or FCDB for their purposes, which can further be used to obtain accurate results in research, education, and in decision making for policy and programming (16). Not only is NutriBase a useful tool to help domain experts compare different datasets and therefore select the most appropriate one, it can also help national compilers to evaluate their own original data and metadata, and ensure the quality datasets. Moreover, an advantage of the system is also that food manufacturers can gain direct access, and add or edit food-related data of their products. In this way, important information about branded foods currently available in stores can be regularly updated and shared with consumers.

The usage of FCDBs may be significantly restricted due to the missing data (3). It has been proposed that it is better to include imputed data, transparently identified as such, than no data at all (3). However, data should only be borrowed or imputed among the same or similar foods. Several computational methods for missing data imputation within FCDBs have been previously researched (20, 21). All of them concluded that, in order to ‘borrow’ data, as many details as possible about the origin or source of the food are needed. In addition, when borrowing data, it is necessary to check whether the relevant values (e.g., nutrients) and metadata are similar. If the metadata or values deviate too much, the foods should not be linked, and a better match should be identified. Deviations may occur for various reasons, such as; different food origin, different analytical methods used or outdated data. The developed DKBMS may ease the process of comparing FCD among different datasets or resources, and help finding the best matches.

Although connecting data from just two FCDBs would be the easiest for compilers, it is not always feasible because different FCDBs contain different data. For example, all of the imported FCDBs contain data for the *total protein* content, but only three FCDBs provide data for specific amino acids. However, research suggests that emphasis should be given not only to the overall protein intake, but also to the specific amino acids [i.e., leucine in older adults, as it is proposed to prevent and treat sarcopenia (49)]. Thus, for experts to prepare dietary guidelines that focus also on specific amino acids and further disseminate them, FCDBs must first contain such information. Among the FCDBs currently imported into NutriBase, only the Danish, Australian, and American FCDBs provide data for leucine, for example. Currently, many imported FCDBs calculate the protein content of foods using a 6.25 nitrogen-to-protein conversion factor as the default factor. However, recent research suggests using specific conversion factors for different foods (50). The new factors and/or re-calculations of protein content can be updated when available and borrowed across FCDBs. Clearly, the DKBMS could also be used to identify globally missing data within the FCDBs.

Nowadays many web-based and mobile applications allow users to add or edit FCD without considering data standards. This may lead to imprecise data, which can further lead to incorrect dietary intake assessments. This is concerning because it raises the question: how can users be sure the data is of high quality? Hence, it is recommended that apps use FCD from approved and high-quality FCDBs, as these guarantee harmonized, scientifically collected, and reviewed data and information. Within the NutriBase, the data origin/source is clearly displayed, and traceability of it is enabled. Combining such trustworthy FCDB with all relevant KBs and sematic resources, can provide a baseline for other systems (e.g., mobile apps, web-based tools, online grocery stores), and it is an extension of what has been done in the past.

In addition, the created KB can be updated by adding and importing direct links to more relevant resources. Some examples of KBs and knowledge resources that could be added to the system are; the international network of food data systems - INFOODS [4], the Global Dietary Database (51), the chemical hazard database (52), different EFSA guidelines, standards and tools (53), etc. Uniting, linking and regularly updating all these resources, could present a baseline for experts and consumers by providing them with transparent, detailed and evidence-based food and nutrition D&K.

Despite the contributions of the current study, the limitations need acknowledgment. As already mentioned, some tasks had to be performed manually, which can be very tedious and usually requires the work of several people. Standardizing and harmonizing D&K among different research fields would allow us to avoid the manual work and expedite the process. In addition, currently only FoodEx2 coding system is implemented in the DKBMS. However, more coding systems could be imported to improve interoperability. Furthermore, although the tool’s user interface is designed to be multilingual, it is currently available only in Slovenian, and not all parts of the tool have been translated into English yet. A complete translation of the tool would allow better distribution among different countries. Moreover, more expert users would need to use and test NutriBase to provide a more comprehensive evaluation. Lastly, while the development of the tool is based on research work, ongoing maintenance and upgrades will require additional and continuous financial support.

### 3.3 Future work

The development of NutriBase demonstrates the complexity of the food compilation process. It shows that many activities have to be performed to develop and maintain high-quality D&K, and to construct the semantic resources needed for the automation of specific steps. The results of the manually performed work presented in the current paper could serve as input for FNS-Harmony. Additionally, new computer-based methodologies to support our future work have been developed, and some solutions have already been implemented as openly available web services (e.g., through the FNS-Cloud catalog [36]). In order to speed up the compilation process, Ispirova et al. (54) developed the methodology for automatic identification of different names of the same foods or dishes (e.g., eggplant and aubergine).

To enable rapid upgrades of D&K, the tool will be integrated within existing or developing knowledge graphs [e.g., FoodKG on food recommendations, FooDB, knowledge graphs on food-disease and food-chemical relations (31, 55), and a knowledge graph on food consumer knowledge being under development within the COMFOCUS project (30)]. Since NutriBase is designed to integrate data with knowledge that is formalized with respect to standardized semantic resources, the connection with any healthcare information system compliant with the openEHR standard (56) is possible. Furthermore, for branded foods and recipes using branded foods as ingredients, the algorithm to calculate values for components that are not mandatory to be included on the nutrition declaration table, can be implemented by using the food matching web services developed within FNS Cloud (57).

Moreover, current FCDBs imported into NutriBase will be updated with the latest releases found, and additional FCDBs may be added. Complementing a FCDB with generic food images would also be beneficial; however, a database of standardized images for generic foods is currently lacking. Having such a database and linking it to FCDBs would facilitate food identification within the FCDBs and support research focusing on automated food image recognition (58). This could further assist in dietary intake assessments and portion size estimations, especially if measurement aids [e.g., (59)] are included.

## 4 Conclusion

The tool called NutriBase presented in the current paper is a comprehensive system that includes not only multiple FCDBs, but also KBs. Combining FCD with relevant knowledge is an extension of what has already been done in this research area. Moreover, all D&K imported are harmonized and compiled with respect to various well-established standards. NutriBase can help create and maintain the quality management system needed to ensure data quality. Merging quality management systems with data production and compilation management enhances the monitoring and assessment of FCDBs, thereby increasing their credibility among consumers, experts, policymakers, and other stakeholders. Additionally, using NutriBase reduces the time required to review FCD by enabling users to add, edit, link, and integrate data with knowledge, all in one place. Domain experts who evaluated and validated the tool would recommend using the system and believe that it is a very usable tool (SUS score 78.9). Moreover, NutriBase represents an important step in transparently borrowing imputed data, and therefore reducing missing data. Lastly, the system is highly modifiable and can be further customized to meet different requirements at the national or international level. Existing and newly generated D&K can be continuously added as long as they comply with standards, which would strengthen the tool even more.

## Data Availability

The data analyzed in this study is subject to the following licenses/restrictions: in the paper an infrastructure in a form of a new data- and knowledge base management system is presented, thus no dataset was generated. The management system is not yet publicly available. Requests to access these datasets should be directed to eva.valencic@ijs.si.
